# Organ‐Specific Phytochemical Composition and Bioactivity Profiling of *Chaerophyllum aksekiense*: A Multiassay Antioxidant, Enzyme Inhibition, and Correlation‐Based Evaluation

**DOI:** 10.1002/open.70220

**Published:** 2026-04-28

**Authors:** Bedrettin Selvi, Elif Aktürk Bozdemir

**Affiliations:** ^1^ Faculty of Arts and Sciences Department of Biology Tokat Gaziosmanpaşa University Tokat Türkiye; ^2^ Vocational School of Organized Industrial Zone Department of Chemistry and Chemical Processing Technologies Tokat Gaziosmanpaşa University Tokat Türkiye

**Keywords:** antioxidant assays, *Chaerophyllum aksekiense*, enzyme inhibition, phytochemical composition, relative antioxidant capacity index

## Abstract

Understanding organ‐specific phytochemical variation is essential for elucidating how chemical diversity shapes biological activity in narrowly distributed Apiaceae. This study provides the first comprehensive evaluation of the nonvolatile phytochemical composition, antioxidant potential, and enzyme inhibitory properties of ethanol extracts obtained from the leaves, flowers, stems, and roots of *Chaerophyllum aksekiense*. Total phenolic and flavonoid levels differed markedly among organs, with leaves exhibiting the highest phenolic content and flowers the greatest flavonoid accumulation. LC–ESI–MS/MS analysis revealed distinct distribution patterns of key compounds, including hyperoside, gallic acid, caffeic acid, and hydroxybenzoic acids. Antioxidant activity differed across assays, with leaf extracts demonstrating the strongest electron‐transfer and radical‐scavenging capacities in phosphomolybdenum, CUPRAC, FRAP, DPPH, and ABTS assays, reflected by the highest relative antioxidant capacity index (RACI) score. Stem extracts were the most effective metal chelators. Enzyme inhibition activities were similarly differentiated; leaves and roots exhibited the strongest cholinesterase inhibition, roots were most active against α‐amylase, and leaves against α‐glucosidase. Correlation analysis highlighted gallic and caffeic acids as major contributors to both antioxidant and enzyme‐modulatory outcomes. Collectively, these findings emphasize that *C. aksekiense* displays pronounced organ‐specific phytochemical organization that drives its diverse bioactivities, and they identify key organs and compounds with potential value for future mechanistic and application‐oriented studies.

## Graphical abstract

This study reveals the organ‐specific phytochemical specialization of the endemic *Chaerophyllum aksekiense* for the first time. Although leaves exhibit the highest phenolic content and antioxidant capacity, flowers stand out with flavonoid accumulation, and roots demonstrate potent enzyme inhibition. These findings document the species' biochemical diversity, providing a strategic dataset for future nutraceutical and pharmacological applications.

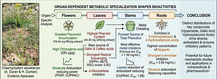

## 1 Introduction

*Chaerophyllum aksekiense* (A. Duran & H. Duman) is an endemic species with a relatively narrow distribution within the Apiaceae family and represents a taxonomically and phytochemically underexplored taxon, the restricted distribution of which makes it particularly relevant for documenting species‐specific metabolite diversity and potential bioactivities. Apiaceae hosts many medicinal and aromatic plants known for their phenolic‐rich compounds and associated potent antioxidant properties. Representatives of this family have been used in both food and folk medicine for centuries; recent research has revealed that this group contains various phenolic acids, flavonoids, and significant antioxidant and antimicrobial activities [1]. The fact that oxidative stress is associated with many chronic diseases [2] makes phenolic‐rich extracts obtained from Apiaceae species important as a source of natural antioxidants or enzyme modulators in the fields of food technology, cosmetic formulations, and drug development.

In this context, when evaluated at the genus level, *Chaerophyllum* comprises approximately 110 species distributed throughout the temperate regions of Asia, Africa, and Europe. Türkiye is represented by 15 species, 4 of which are endemic taxa. Members of the genus are traditionally used in some regions to flavor cheese and dishes; chemically, they are characterized by essential oils, lignans, polyacetylene compounds, phenolic acids, and various flavon derivatives [3]. Indeed, the essential oil contents and biological activities of some *Chaerophyllum* species have been evaluated in detail in the literature. For example, *C. aromaticum* and *C. aureum*, with their complex essential oil profiles, exhibit significant antioxidant, antimicrobial, and cholinesterase inhibitory effects, whereas *C. byzantinum* leaves have been reported to exhibit high phenolic accumulation and strong in vitro antioxidant capacity [4, 5, 6]. These findings indicate that the genus is still a poorly investigated group, although it holds potential for biologically valuable metabolites.

*C. aksekiense* is a young species identified from calcareous rocky habitats in Southern Türkiye, and its distribution is limited to high‐altitude areas in the Akseki region [7]. Research on the species has so far largely focused on its essential oil composition; the only available study analyzed the components of the essential oil obtained from crushed fruits, showing a predominance of compounds such as heptacosane and caryophyllene oxide [3]. Although caryophyllene oxide has been reported to exhibit moderate acetylcholinesterase inhibitory activity, and essential oils rich in long‐chain hydrocarbons, including heptacosane, may show some antimicrobial potential despite relatively low radical‐scavenging capacity, these volatile constituents do not adequately explain the broader antioxidant and multienzyme inhibitory responses evaluated in the present study [8, 9]. Therefore, although the previously reported essential oil composition offers a useful starting point for the chemical characterization of the species, it remains insufficient for understanding the phenolic‐based antioxidant and enzyme‐modulatory properties of organ‐specific ethanol extracts. Therefore, the nonvolatile chemical fraction and redox‐based biological activity of *C. aksekiense* remain completely unclear, creating a significant gap both for intrageneric assessments and for future research on regional Apiaceae endemics, particularly in understanding how organ‐level phytochemical variation translates into functional biological responses such as enzyme inhibition and antioxidant capacity.

Current studies indicate that phenolic content and antioxidant capacity can vary significantly among plant organs, often influenced by developmental stages and microhabitat conditions. Organ‐based studies in other species indicate that, in some cases, phenolic compounds with high reducing power are concentrated in the belowground parts, whereas flavonoids with strong radical‐scavenging activity may dominate aerial parts such as leaves and flowers [10]. Identifying such variation is important not only for identifying the most biologically active organs but also for developing chemotaxonomic indicators that can be used to distinguish closely related species. However, there are no data on differences in phenolic composition or antioxidant response between organs specific to *C. aksekiense*.

This study was conducted to investigate the phenolic profiles, antioxidant, and enzyme inhibitory properties of extracts obtained from different organs of *C. aksekiense* with an integrative approach, with the specific aim of elucidating organ‐dependent functional specialization and providing a comprehensive biochemical dataset for a previously uncharacterized endemic species. Ultrasound‐assisted ethanol extracts were prepared from leaf, stem, flower, and root tissues; total phenolic and flavonoid levels were measured by spectrophotometric methods; and selected phenolic compounds were identified by LC–ESI–MS/MS. Antioxidant capacity was assessed using a broad panel of radical scavenging, reducing power, phosphomolybdenum total antioxidant capacity, and Fe(II) chelation tests. The extracts were also tested for inhibitory activities on cholinesterases (ChEs), α‐amylase, α‐glucosidase, and tyrosinase. Because no data are available in the literature, all phytochemical and biological activity findings reported in this study are presented for the first time for *C. aksekiense*, thereby generating baseline knowledge that enables comparison with other *Chaerophyllum* species and contributes to chemotaxonomic and bioactivity‐driven research frameworks. This study, which documents variations in phenolic content and antioxidant response among organs, provides an initial dataset that will form the basis for future chemotaxonomic, ecological, and application‐oriented research and offers insight into how organ‐specific metabolite distribution may influence enzyme‐targeted biological activities relevant to pharmacological and nutraceutical applications.

## 2 Materials and Methods

### 2.1 Plant Material

Specimens of *C. aksekiense* were collected during the full bloom period on limestone cliffs in Pınarbaşı village of Akseki district of Antalya on June 18, 2025 (37°47′15″N, 31°49′8″E; 1700–1800 m). The species identification was made by Dr. Bedrettin Selvi, and the herbarium specimen numbered GOPU 9629 was recorded in the Herbarium of Tokat Gaziosmanpaşa University, Faculty of Arts and Sciences. In the study, four organs of the plant, namely, leaf, stem, flower, and root, were used after being separated from each other during the collection stage.

### 2.2 Ethanol Extraction

The collected plant material was air‐dried under shade in a well‐ventilated environment with low humidity and away from direct sunlight at room temperature (approximately 25°C ± 2°C) for 5–7 days until constant weight was achieved. Although the temperature was not actively controlled, it remained within standard laboratory conditions (22°C–25°C). The dried samples were then ground into a fine powder using a blender.

Ultrasound‐assisted extraction was applied for each organ; extraction was carried out in a sonication bath for 1 h using absolute ethanol (99%, v/v) with a 1:20 (w/v) sample‐to‐solvent ratio [11]. This solvent was selected to enable the extraction of a broad range of phytochemicals, including both moderately polar and less polar constituents. Although aqueous ethanol mixtures are often reported to enhance the extraction efficiency of highly polar phenolics, high‐concentration ethanol has also been widely employed to obtain extracts with diverse chemical profiles suitable for bioactivity screening [12, 13].

The resulting ethanol extracts were concentrated under vacuum in a rotary evaporator and stored at 4°C. The yields of *C. aksekiense* flower, leaf, stem, and root extracts were determined as 14.11%, 16.06%, 4.06%, and 4.48%, respectively.

### 2.3 Determination of the Phenolic Compositions of the Extracts

Total phenolic and flavonoid contents of the extracts were analyzed by spectrophotometric methods [14, 15, 16]. The determination of individual phenolic compounds was performed using a previously validated LC–ESI–MS/MS method [17]. Quantitative analysis was carried out on an Agilent 1260 Infinity LC system coupled to a 6420 Triple Quadrupole mass spectrometer operating in multiple reaction monitoring (MRM) mode, which provides high selectivity and sensitivity for targeted phenolic profiling. Chromatographic separation was achieved on a Poroshell 120 EC‐C18 column using a gradient system consisting of 0.1% formic acid (solvent A) and methanol (solvent B).

Method validation parameters were evaluated on the basis of linearity, sensitivity, and reproducibility. Calibration curves for all analytes showed excellent linearity (*R* ^2^ ≥ 0.99), whereas limit of detection (LOD) and limit of quantification (LOQ) confirmed adequate sensitivity for trace‐level determination. Compound identification was based on retention time matching and characteristic precursor/product ion transitions compared with authentic standards analyzed under identical conditions. These validation parameters ensure the reliability and quantitative accuracy of the LC–MS/MS measurements. Detailed analytical characteristics (MRM transitions, calibration equations, LOD and LOQ values) are additionally provided in Tables S1 and S2 for reference.

### 2.4 Biological Activity

Antioxidant assays, including phosphomolybdenum, DPPH, ABTS, CUPRAC, FRAP, and metal‐chelating activity, were performed according to previously reported methods [14, 18, 19, 20, 21, 22] with minor adaptations. Enzyme inhibitory activities against acetylcholinesterase (AChE), butyrylcholinesterase (BChE), tyrosinase, α‐amylase, and α‐glucosidase were evaluated following established protocols [23, 24, 25].

Briefly, antioxidant assays were based on either electron‐transfer mechanisms (CUPRAC, FRAP, phosphomolybdenum) or radical scavenging capacity (DPPH, ABTS), whereas metal‐chelating activity was assessed using ferrozine‐based complex formation. Enzyme inhibition assays were conducted using spectrophotometric methods in microplate format, and all measurements included appropriate blanks and negative controls for background correction.

For all bioactivity assays, concentration–response curves were constructed using at least five different extract concentrations, and IC_50_ or EC_50_ values were calculated as the concentration required to achieve 50% inhibition or activity. These values were obtained by nonlinear regression analysis of the dose–response curves using appropriate software. This approach ensures a quantitative and comparable evaluation of extract potency across different assays.

Reference standards (Trolox, EDTA, galanthamine, kojic acid, and acarbose) were analyzed under the same experimental conditions to allow direct comparison of activity levels. Additional procedural details and full experimental conditions are provided in the Supporting Information section.

In all assays, appropriate blank and negative control measurements were used for background correction and calculation of activity, whereas the activities of the extracts were evaluated against the corresponding reference standards reported.

### 2.5 Statistical Analysis

All results are expressed as mean ± standard deviation (SD) of three independent experiments (*n* = 3). Statistical significance was evaluated using one‐way analysis of variance (ANOVA) followed by Tukey's post hoc test, with significance set at *p* < 0.05. Analyses were performed using SPSS 26.0 software. Significant differences between groups are indicated in tables and figures by different superscript letters according to Tukey's HSD test.

Pearson's correlation test was used to examine the relationships between variables. Because antioxidant assays have different mechanisms of action, direct comparison of results was not appropriate. Therefore, the relative antioxidant capacity index (RACI) was calculated to ensure consistent assessment across assays. RACI values were standardized according to the mean and SD of each assay. Additionally, correlations between RACI and individual antioxidant assays were analyzed [26].

### 2.6 Use of Artificial Intelligence (AI)

AI tools were used solely to enhance the linguistic quality of the text, ensure conceptual clarity, and strengthen the overall presentation of the manuscript. All experimental work, laboratory applications, and data generation were performed entirely by the authors; AI was not involved in the processes. AI support for the text was limited to wording editing, and all AI suggestions were carefully reviewed and approved by the authors for scientific consistency and research ethics.

## 3 Results and Discussion

### 3.1 Chemical Composition

**FIGURE 1:**
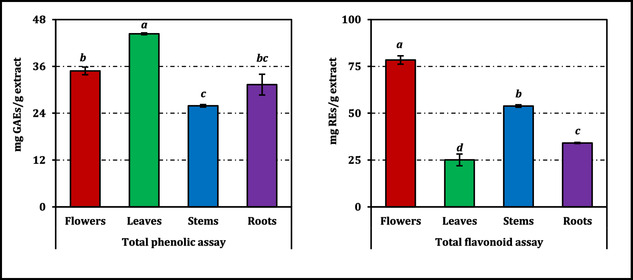
Total phenolic and flavonoid contents of *C. aksekiense* extracts. GAEs and REs: Gallic acid and rutin equivalents, respectively. Values indicated by the same superscripts (a–d) within the same column are not significantly different according to Tukey's HSD test at the 5% significance level.

Total phenolic and flavonoid contents varied markedly among the organ‐specific ethanol extracts of *C. aksekiense* (Figure 1). Leaves exhibited the highest total phenolic content, reaching 44.36 mg GAEs/g extract and forming a statistically distinct group from the other organs (*p* < 0.05). Flowers and roots showed intermediate phenolic levels (34.84 and 31.33 mg GAEs/g extract, respectively), whereas stems were the poorest source of total phenolics with 25.91 mg GAEs/g extract, with the overall differences among organ groups being significant (*p* < 0.05). In contrast, the distribution pattern of total flavonoids was reversed: Flowers contained by far the highest flavonoid content (78.44 mg REs/g extract), followed by stems (53.84 mg REs/g), whereas roots (34.13 mg REs/g) and especially leaves (25.09 mg REs/g) were comparatively depleted, and these differences were also statistically significant (*p* < 0.05).

Targeted LC–ESI–MS/MS analysis further revealed pronounced qualitative and quantitative differences in individual phenolic constituents among the extracts (Table 1).

**TABLE 1:** Concentration (µg/g extract) of selected phenolic compounds in *C. aksekiense* extracts.

No	Compounds	Flowers	Leaves	Stems	Roots
1	Hyperoside	706 ± 16^b^	497 ± 4^c^	2337 ± 17^a^	137 ± 1^d^
2	Chlorogenic acid	283 ± 9^a^	66.4 ± 0.2^d^	181 ± 1^c^	252 ± 1^b^
3	Vanillin	nd	6.4 ± 0.2^c^	68.5 ± 1.2^a^	64.2 ± 0.3^b^
4	Ferulic acid	15.2 ± 0.4^c^	13.1 ± 0.3^d^	54.2 ± 0.4^a^	20.9 ± 0.6^b^
5	3‐Hydroxybenzoic acid	48.0 ± 0.1^b^	33.1 ± 0.1^d^	44.6 ± 0.1^c^	69.0 ± 0.6^a^
6	*p*‐Coumaric acid	30.3 ± 0.2^c^	33.6 ± 0.2^b^	43.4 ± 0.9^a^	43.1 ± 0.2^a^
7	Quercetin	64.1 ± 2.7^a^	13.1 ± 0.1^c^	43.1 ± 0.5^b^	8.86 ± 0.31^c^
8	4‐Hydroxybenzoic acid	47.0 ± 0.2^b^	32.2 ± 0.9^d^	42.0 ± 0.2^c^	67.0 ± 0.7^a^
9	Syringic acid	11.7 ± 0.2^c^	12.6 ± 0.5^c^	36.1 ± 0.4^a^	31.8 ± 0.8^b^
10	Protocatechuic acid	12.1 ± 0.1^c^	12.8 ± 0.2^c^	33.2 ± 0.3^a^	23.2 ± 0.6^b^
11	Hesperidin	4.11 ± 0.31^c^	1.78 ± 0.01^d^	23.5 ± 0.7^a^	5.82 ± 0.27^b^
12	Verbascoside	9.12 ± 0.04^b^	4.33 ± 0.13^d^	11.0 ± 0.1^a^	7.72 ± 0.20^c^
13	Gallic acid	5.02 ± 0.42^d^	26.9 ± 0.2^a^	10.4 ± 0.1^b^	8.95 ± 0.20^c^
14	Caffeic acid	6.46 ± 0.18^d^	28.5 ± 0.1^a^	9.79 ± 0.31^c^	13.9 ± 0.2^b^
15	Rosmarinic acid	4.89 ± 0.05^c^	2.41 ± 0.04^d^	8.31 ± 0.23^b^	19.5 ± 0.4^a^
16	Apigenin	nd	0.63 ± 0.02^c^	5.30 ± 0.14^b^	46.2 ± 0.2^a^
17	(−)‐Epicatechin	nd	nd	nd	nd
18	(+)‐Catechin	nd	nd	nd	nd
19	2‐Hydroxycinnamic acid	nd	nd	nd	nd
20	3,4‐Dihydroxyphenylacetic acid	nd	nd	nd	nd
21	Apigenin 7‐glucoside	nd	nd	nd	nd
22	Eriodictyol	nd	nd	nd	nd
23	Kaempferol	nd	nd	nd	nd
24	Luteolin 7‐glucoside	nd	nd	nd	nd
25	Luteolin	nd	nd	nd	nd
26	Pinoresinol	nd	nd	nd	nd
27	Sinapic acid	nd	nd	nd	nd
28	Taxifolin	nd	nd	nd	nd

*Note:* Values are expressed as mean ± standard deviation (SD) of three independent experiments (*n* = 3). Values indicated by the same superscripts (a–d) within the same row are not significantly different according to Tukey's HSD test at the 5% significance level.

Abbreviation: nd, not detected.

The identification and quantification of phenolic compounds were performed using a validated LC–ESI–MS/MS method operating in MRM mode. Compound identification was based on a combination of retention time matching, precursor ion ([M–H]^−^/[M+H]^+^), and characteristic product ion transitions obtained from MS/MS fragmentation patterns. These parameters were directly compared with those of authentic reference standards analyzed under identical chromatographic conditions. The use of MRM mode ensured high selectivity and sensitivity, allowing reliable discrimination of structurally similar phenolic compounds. The identification strategy is consistent with widely accepted LC–MS/MS practices for phenolic profiling, where both chromatographic and mass spectrometric criteria are required for reliable compound assignment. Detailed MS/MS parameters, including retention times, precursor ions, and fragment ions, as well as calibration data (linearity, LOD, LOQ), are provided in Tables S1 and S2. Representative chemical structures of the major phenolic constituents identified in this study are provided in Figure S1 to facilitate structural interpretation and to highlight the diversity of phenolic acids, flavonoids, and phenylpropanoid derivatives detected.

Stems generally accumulated the highest amounts of several key compounds, most notably hyperoside (2337 µg/g extract), along with elevated levels of ferulic acid, syringic acid, protocatechuic acid, hesperidin, verbascoside, and *p*‐coumaric acid. Roots, in turn, were particularly enriched in 3‐hydroxybenzoic acid (69.0 µg/g), 4‐hydroxybenzoic acid (67.0 µg/g), and rosmarinic acid (19.5 µg/g) and uniquely contained the highest concentration of apigenin (46.2 µg/g extract). Flowers were characterized by comparatively high amounts of chlorogenic acid (283 µg/g) and quercetin (64.1 µg/g), whereas leaves stood out as the main source of gallic (26.9 µg/g) and caffeic acids (28.5 µg/g). Vanillin was absent in flowers but present in the other organs, with stems and roots showing similarly high levels.

The present study offers the first comprehensive account of the nonvolatile phytochemical profile of *C. aksekiense*, thereby filling a critical gap in the chemical characterization of this narrowly distributed endemic. Prior research on the species has been confined exclusively to its essential oil composition, which revealed various monoterpenes and sesquiterpenes but provided no insight into its phenolic or flavonoid constituents [27]. By contrast, the organ‐specific ethanol extracts examined here demonstrated a chemically rich and markedly heterogeneous phenolic landscape, underscoring that the biosynthesis and allocation of phenolics within Apiaceae members can vary considerably across plant organs, as also emphasized by Tel‐Çayan et al. [28].

The observed organ‐dependent partitioning of phenolics and flavonoids in *C. aksekiense* aligns with earlier findings from other *Chaerophyllum* taxa, which likewise exhibit pronounced chemical differentiation among tissues. The higher total phenolic content observed in leaves can be mechanistically associated with their direct exposure to environmental stressors such as ultraviolet radiation, oxidative pressure, and herbivory, which are known to induce the phenylpropanoid pathway and promote the accumulation of phenolic acids with strong redox‐buffering capacity [29]. In contrast, the predominance of flavonoids in flowers is likely linked to their functional roles in pigmentation, UV filtration, and pollinator attraction, as flavonoid subclasses such as flavonols and anthocyanin precursors contribute to visual signaling and reproductive success [30]. This differential allocation reflects a functional specialization in secondary metabolism, where leaves prioritize protective antioxidant phenolics, whereas flowers allocate more resources toward flavonoid biosynthesis associated with reproductive ecology. Such organ‐specific metabolic differentiation is not unique to this species but appears to be a broader characteristic within the genus *Chaerophyllum*.

For example, roots of *C. hirsutum* were previously shown to accumulate lignans, coumarins, phenylpropanoids, and polyacetylenes—including novel lignan structures—illustrating the metabolic specialization of subterranean organs [31]. Similarly, subaerial parts of *C. aureum* were reported to contain an array of lignans and phenylpropanoids not uniformly distributed across tissues [32]. The presence of diverse phenolic acids, flavonoids, and glycosidic derivatives in *C. macropodum* likewise reinforces the notion that phenolic metabolism in the genus is neither uniform nor organ‐independent [33].

Several of the compounds highlighted in the present work—such as chlorogenic acid, rosmarinic acid, ferulic acid, and various flavonoid glycosides—have also been documented in other *Chaerophyllum* species [28, 34], situating *C. aksekiense* within the broader chemotaxonomic patterns known for the genus. Nevertheless, the distributional patterns detected here, including the selective enrichment of specific phenolics in stems, roots, leaves, or flowers, illustrate a level of intraplant metabolic differentiation not previously reported for *Chaerophyllum*. Because nearly all published work on the genus has focused on volatile constituents, the present dataset substantially broadens the chemical dimension through which *Chaerophyllum* species can be compared.

Importantly, the detection of multiple structurally diverse metabolites—ranging from hydroxybenzoic and hydroxycinnamic acids to flavonols, flavones, and glycosylated derivatives—suggests that *C. aksekiense* may harbor untapped chemotaxonomic and bioactive potential. The coexistence of compounds such as gallic acid, hyperoside, quercetin, verbascoside, and apigenin mirrors the chemical complexity described for species like *C. bulbosum* and *C. villosum*, which were reported to contain overlapping yet distinct phenolic repertoires [28, 35]. These parallels strengthen the view that nonvolatile phenolics constitute a meaningful but underexplored dimension of diversity within the genus.

This study not only establishes the first detailed nonvolatile phenolic profile of *C. aksekiense* but also contributes a valuable comparative framework for future investigations on *Chaerophyllum*. By documenting clear organ‐specific chemical signatures and identifying several compounds with known biological relevance, the findings open new avenues for chemotaxonomic studies, bioactivity‐guided fractionation, and conservation‐oriented phytochemical research. Further metabolomic and biosynthetic studies, ideally integrating environmental and microhabitat variables, would help clarify the drivers of phenolic variability both within and among *Chaerophyllum* species.

### 3.2 Antioxidant Activity

The antioxidant responses of *C. aksekiense* extracts varied markedly across assays (Table 2), with statistically significant differences among organs in each assay (*p* < 0.05). In electron‐transfer‐based tests, the leaves consistently exhibited the strongest performance among plant parts, as reflected by their consistently lower EC_50_ values across all electron‐transfer‐based assays, indicating higher antioxidant potency. However, all extracts remained less potent than the corresponding standard Trolox, the EC_50_ values of which were markedly lower in phosphomolybdenum (0.45 mg/mL), CUPRAC (0.19 mg/mL), and FRAP (0.05 mg/mL) assays (Table 2). This trend was pronounced in the phosphomolybdenum assay, where the leaves showed the lowest EC_50_ (1.11 mg/mL), followed by stems and roots, whereas flowers exhibited the weakest activity and was further supported by CUPRAC and FRAP results, with EC_50_ values of 0.91 and 0.46 mg/mL, respectively, again confirming the superior reducing capacity of leaf extracts based on lower effective concentrations. Flowers displayed moderate efficiencies across the same assays, whereas stems and roots showed comparatively weaker reducing capacities, particularly in the CUPRAC test, where the stems required the highest concentration (1.88 mg/mL).

**TABLE 2:** Antioxidant activities of *C. aksekiense* extracts.

Assays	Flowers	Leaves	Stems	Roots	Trolox	EDTA
Phosphomolybdenum (EC_50_: mg/mL)	1.63 ± 0.07^d^	1.11 ± 0.07^b^	1.38 ± 0.01^c^	1.41 ± 0.04^c^	0.45 ± 0.04^a^	—
CUPRAC‐reducing power (EC_50_: mg/mL)	1.39 ± 0.02^c^	0.91 ± 0.03^b^	1.88 ± 0.03^e^	1.51 ± 0.04^d^	0.19 ± 0.01^a^	—
FRAP‐reducing power (EC_50_: mg/mL)	0.62 ± 0.02^c^	0.46 ± 0.01^b^	0.82 ± 0.02^d^	1.43 ± 0.01^e^	0.05 ± 0.01^a^	—
DPPH radical (IC_50_: mg/mL)	5.40 ± 0.56^c^	2.83 ± 0.07^b^	7.99 ± 0.16^d^	16.36 ± 0.28^e^	0.27 ± 0.02^a^	—
ABTS radical cation (IC_50_: mg/mL)	2.31 ± 0.09^c^	1.56 ± 0.16^b^	2.72 ± 0.19^cd^	3.14 ± 0.12^d^	0.20 ± 0.02^a^	—
Ferrous ion chelating (IC_50_: mg/mL)	2.02 ± 0.04^c^	3.60 ± 0.18^e^	1.36 ± 0.01^b^	2.93 ± 0.07^d^	—	0.02 ± 0.002^a^

*Note:* Values are expressed as mean ± standard deviation (SD) of three independent experiments (*n* = 3). Values indicated by the same superscripts (a–e) within the same row are not significantly different according to Tukey's HSD test at the 5% significance level.

Abbreviation: EDTA, ethylenediaminetetraacetic acid (disodium salt).

**FIGURE 2:**
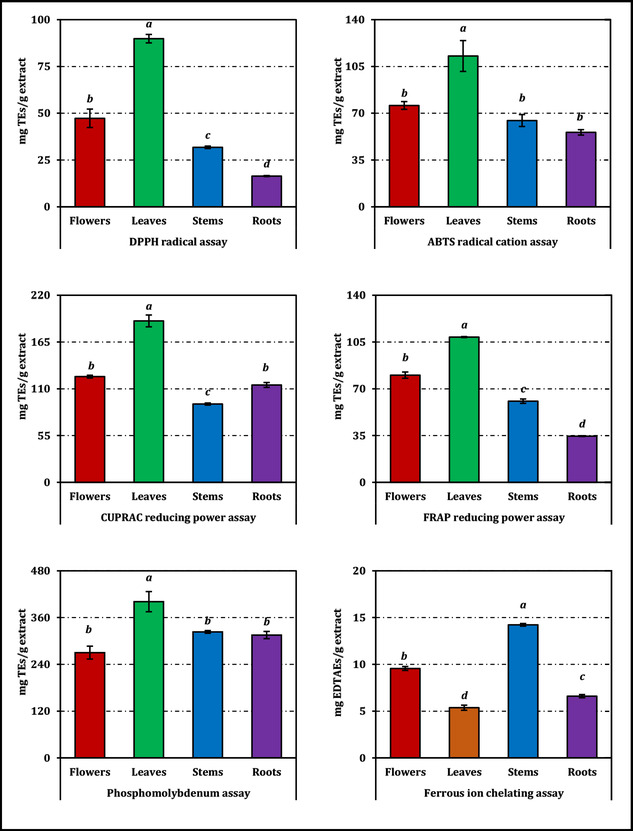
Antioxidant activity of *C. aksekiense* extracts. TEs and EDTAEs, Trolox and ethylenediaminetetraacetic acid (disodium salt) equivalents, respectively. The activities of the extracts are expressed relative to the corresponding reference standards, the IC_50_/EC_50_ values of which are additionally provided in Table 2 for direct comparison. Values indicated by the same superscripts (a–d) on the bar chart are not significantly different according to Tukey's HSD test at the 5% significance level.

A similar organ‐dependent pattern emerged in radical‐scavenging assays. Leaves again demonstrated the highest scavenging ability, achieving IC_50_ values of 2.83 mg/mL (DPPH) and 1.56 mg/mL (ABTS), which were substantially lower than those of flowers, stems, and especially roots, indicating a markedly higher radical‐scavenging efficiency, distinctly outperforming flowers, stems, and especially roots, which were the least responsive in both tests (16.36 and 3.14 mg/mL, respectively), and these differences were significant at *p* < 0.05. Nevertheless, Trolox remained clearly more potent than all extracts in both assays, with IC_50_ values of 0.27 mg/mL for DPPH and 0.20 mg/mL for ABTS (Table 2). Metal‐chelating activity followed a different distribution: The stems produced the most effective ferrous ion sequestration (IC_50_ = 1.36 mg/mL), representing the lowest IC_50_ among organs and therefore the highest chelating efficiency, whereas the leaves and roots showed limited chelating potential, again with significant interorgan variation (*p* < 0.05). Even so, the chelating activity of all extracts was markedly weaker than that of EDTA (IC_50_ = 0.02 mg/mL; Table 2). Complete activity profiles expressed as Trolox or EDTA equivalents can be additionally consulted in Figure 2.

**FIGURE 3:**
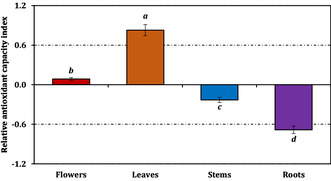
Relative antioxidant capacity index of *C. aksekiense* extracts. Values indicated by the same superscripts (a–d) on the bar chart are not significantly different according to Tukey's HSD test at the 5% significance level.

**FIGURE 4:**
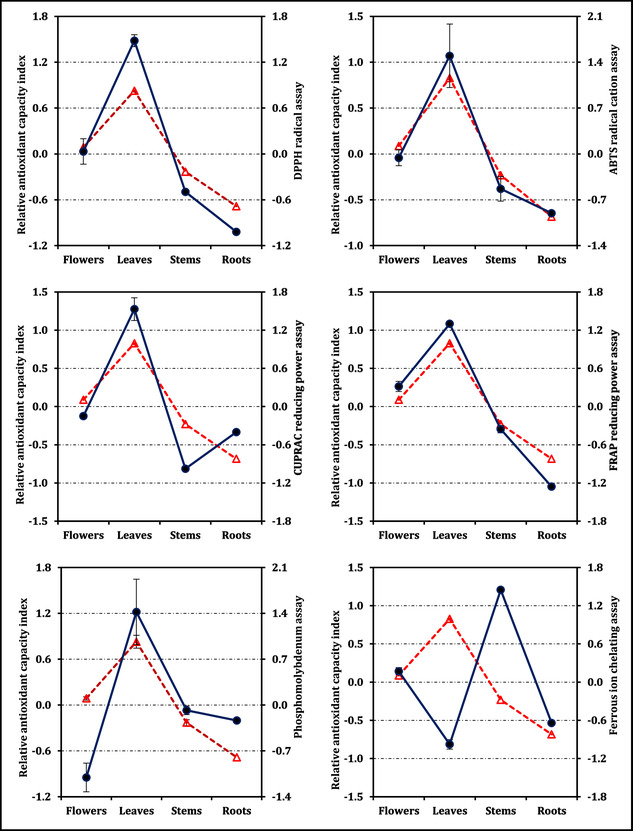
Correlation between the relative antioxidant capacity index (dashed red line with triangle) and antioxidant activity (solid dark blue line with circle).

The overall antioxidant capacities synthesized through the RACI mirrored these assay‐specific outcomes (Figure 3). Leaves reached the highest composite value (0.83), followed by flowers (0.09), whereas stems (–0.23) and roots (–0.68) clustered at the lower end, with significant differences among groups as indicated in the figure (*p* < 0.05). Correlation analysis (Figure 4) revealed that RACI was strongly aligned with all antioxidant assays except ferrous ion chelation, for which leaves and stems exhibited an inverse relationship between their IC_50_ values and respective RACI scores.

The present study provides the first comprehensive account of the antioxidant properties of *C. aksekiense*, thereby filling a significant gap in the phytochemical characterization of this narrowly distributed taxon. The marked assay‐dependent and organ‐specific variations observed here are broadly consistent with patterns previously reported for congeners. Studies on *C. macropodum* and *C. bulbosum*, for instance, highlight that antioxidant potential in the genus is strongly influenced by extraction solvent, plant part, and chemical composition [28, 33]. Our findings extend this trend to *C. aksekiense*, revealing clear superiority of leaf extracts in electron‐transfer and radical‐scavenging reactions, whereas stems were comparatively more competent in metal‐chelating activity. These observations are in line with previous studies on *Chaerophyllum* species, where phenolic‐rich extracts were reported to exhibit stronger antioxidant activity than less polar fractions, and where significant differences between plant parts were attributed to variations in phenolic composition [33, 36].

A key comparative dimension concerns the contrast between extract‐based and essential‐oil‐based antioxidant profiles. Several *Chaerophyllum* species exhibit weak or negligible antioxidant activities in their essential oils, despite demonstrable efficacy in polar extracts. This phenomenon has been documented for *C. macropodum* [37], *C. libanoticum* [38], *C. aromaticum* [39], and *C. villosum* [35].

The consistently low radical‐scavenging capacities of essential oils across these studies underscore the dominance of nonvolatile phenolic constituents in shaping antioxidant performance. The strong activities recorded for *C. aksekiense* leaf and flower extracts therefore align well with this chemotaxonomic pattern, further supporting the interpretation that phenolic richness—rather than volatile composition—is the principal determinant of antioxidant competence in the genus. This interpretation is additionally reinforced by outcomes from *C. macropodum*, where methanolic extracts outperform aqueous extracts and essential oils, a trend mirrored by the efficient radical scavenging observed in our study [33, 37].

The organ‐specific divergence detected in *C. aksekiense* is also noteworthy. Although leaves displayed the strongest activity in almost all electron‐transfer and radical‐scavenging assays, the stems were uniquely capable of more effective metal chelation. Such complementary functional profiles are frequently encountered in *Chaerophyllum* species. Notably, Tel‐Çayan et al. [28] demonstrated that roots and aerial parts of *C. bulbosum* differ markedly in their antioxidant responses, with some assays favoring root extracts and others favoring aerial tissues. That study also employed chemometric tools to show that phytochemical variation across organs is a major driver of antioxidant differentiation—a conclusion fully compatible with the contrasting assay outcomes we observed among leaves, flowers, stems, and roots of *C. aksekiense*. Additionally, the relatively moderate‐to‐strong antioxidant activity displayed by *C. aksekiense* extracts parallels the activities reported in several *Chaerophyllum* taxa used traditionally as food or medicine. For instance, *C. macropodum* and *Heracleum persicum*—both from Apiaceae—have been recognized as potential natural antioxidants and GST modulators [40], and individual flavonoid glycosides isolated from *C. bulbosum* aerial parts have shown even higher radical‐scavenging capacity than reference antioxidants [36]. These parallels highlight the broader functional versatility of *Chaerophyllum* species and position *C. aksekiense* as a promising candidate for further bioactivity‐guided isolation studies.

### 3.3 Enzyme Inhibitory Activity

**FIGURE 5:**
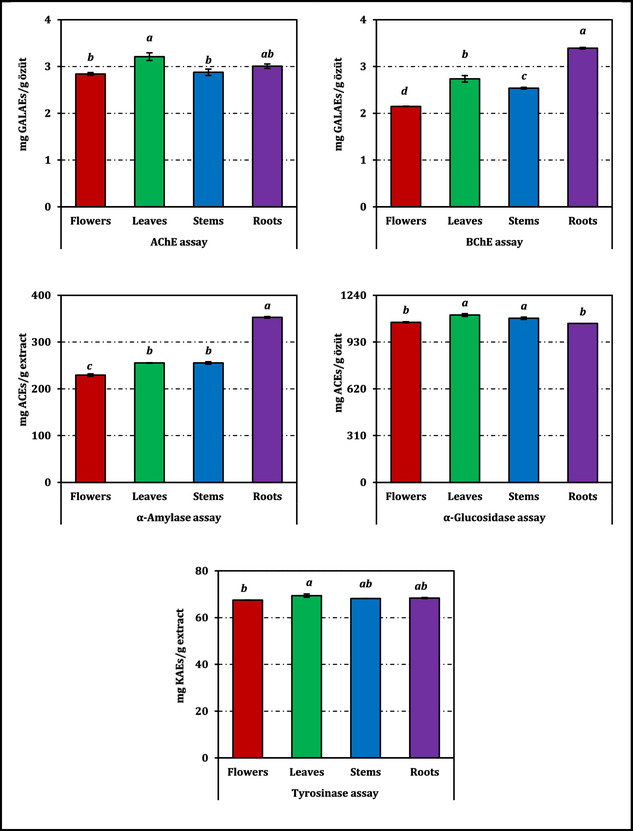
Enzyme inhibition activity of *C. aksekiense* extracts. ACEs, GALAEs, and KAEs mean acarbose, galanthamine, and kojic acid equivalents, respectively. The inhibitory responses of the extracts are expressed relative to the relevant standards, the IC_50_ values of which are reported in Table 3 for direct evaluation of potency. Values indicated by the same superscripts (a–d) on the bar chart are not significantly different according to Tukey's HSD test at the 5% significance level.

The organ‐dependent extracts of *C. aksekiense* exhibited measurable inhibitory effects across all assayed enzymes, and their inhibitory potencies were directly compared on the basis of IC_50_ values, where lower values indicate stronger inhibition, with distinct patterns emerging among plant parts (Table 3), and the interorgan differences were significant for all enzyme assays (*p* < 0.05). For clarity, the activities of the extracts should be interpreted in direct relation to the corresponding positive controls reported in Table 3 and visualized in Figure 5. For ChEs, the leaves displayed the strongest AChE inhibition (IC_50_ = 1.00 mg/mL), indicating the highest inhibitory potency, followed closely by roots (1.06 mg/mL) and stems (1.11 mg/mL), whereas flowers were comparatively weaker (1.13 mg/mL), thus establishing a clear potency ranking of leaves > roots > stems > flowers based on IC_50_ values, with significant differences among the tested organs (*p* < 0.05). A similar organ‐specific trend was observed for BChE, where roots showed the highest potency (0.94 mg/mL), as evidenced by the lowest IC_50_ value, outperforming leaves (1.17 mg/mL), stems (1.26 mg/mL), and flowers (1.49 mg/mL), which exhibited progressively weaker inhibition with increasing IC_50_ values, and these differences were statistically significant (*p* < 0.05). However, all extracts were markedly less potent than galanthamine (IC_50_ = 0.0035 mg/mL for both AChE and BChE), indicating measurable but moderate cholinesterase inhibition at the crude extract level rather than strong activity comparable to the reference inhibitor.

**TABLE 3:** Enzyme inhibition activity of *C. aksekiense* extracts.

Samples	AChE inhibition (IC_50_: mg/mL)	BChE inhibition (IC_50_: mg/mL)	Tyrosinase inhibition (IC_50_: mg/mL)	α‐Amylase inhibition (IC_50_: mg/mL)	α‐Glucosidase inhibition (IC_50_: mg/mL)
Flowers	1.13 ± 0.01^c^	1.49 ± 0.01^e^	1.22 ± 0.01^d^	4.10 ± 0.05^d^	1.07 ± 0.01^ab^
Leaves	1.00 ± 0.03^b^	1.17 ± 0.03^c^	1.19 ± 0.01^b^	3.68 ± 0.01^c^	1.02 ± 0.01^a^
Stems	1.11 ± 0.03^c^	1.26 ± 0.01^d^	1.21 ± 0.01^bc^	3.68 ± 0.04^c^	1.04 ± 0.01^ab^
Roots	1.06 ± 0.02^bc^	0.94 ± 0.01^b^	1.21 ± 0.01^bc^	2.66 ± 0.01^b^	1.08 ± 0.01^c^
Galanthamine	0.0035 ± 0.0003^a^	0.0035 ± 0.0004^a^	—	—	—
Kojic acid	—	—	0.08 ± 0.01^a^	—	—
Acarbose	—	—	—	1.00 ± 0.05^a^	1.16 ± 0.03^d^

*Note:* Values are expressed as mean ± standard deviation (SD) of three independent experiments (*n* = 3). Values indicated by the same superscripts (a–e) within the same column are not significantly different according to Tukey's HSD test at the 5% significance level.

n the tyrosinase assay, the most effective inhibition was recorded for the leaves (IC_50_ = 1.19 mg/mL), whereas the flowers exhibited the lowest activity (1.22 mg/mL). Stems and roots showed nearly identical values (1.21 mg/mL), indicating limited variation among these organs. Compared with kojic acid (IC_50_ = 0.085 mg/mL), all extracts showed much weaker inhibition, suggesting only modest tyrosinase‐modulating potential. Figure 5, which presents results in positive‐control equivalents, further highlights the relative distribution of activities among extracts.

Carbohydrate‐hydrolyzing enzyme inhibition also varied across organs. α‐Amylase inhibition was strongest in the roots (IC_50_ = 2.66 mg/mL), representing the lowest IC_50_ among all organs and therefore the highest inhibitory efficiency, representing a clear advantage over leaves and stems (both 3.68 mg/mL) and flowers (4.10 mg/mL), with significance established at *p* < 0.05. Nevertheless, all extracts were weaker than acarbose in the α‐amylase assay, where the standard showed an IC_50_ of 1.00 mg/mL (Table 3). In contrast, α‐glucosidase inhibition did not follow the same pattern: The most active samples were the leaves (IC_50_ = 1.02 mg/mL) and stems (1.04 mg/mL), the lower IC_50_ values of which indicate stronger inhibition compared to roots and flowers, whereas roots (1.08 mg/mL) and flowers (1.07 mg/mL) showed slightly reduced potency, and the statistical groupings are presented in Table 3 (*p* < 0.05). In the α‐glucosidase assay, all extracts displayed slightly lower IC_50_ values than acarbose (1.16 mg/mL), indicating that their inhibitory effects were comparable to, and in this dataset marginally stronger than, the reference standard. These results support the conclusion that α‐glucosidase inhibition represents one of the more notable bioactivities of the organ extracts, despite the crude nature of the samples.

The present study also provides the first organ‐specific evaluation of enzyme inhibitory properties in *C. aksekiense*, thereby substantially expanding the biochemical knowledge of this narrow endemic taxon. Although no prior investigations exist for this species, the inhibitory patterns documented here align well with the broader trends reported for other *Chaerophyllum* members, particularly in terms of extract‐ and organ‐dependent variability. Comparable enzyme inhibitory profiles have been reported for *C. bulbosum*, where both crude extracts and isolated constituents exhibited activity against cholinesterases, tyrosinase, and carbohydrate‐hydrolyzing enzymes, supporting the pharmacological relevance of the genus [36, 41]. In addition, studies on *C. aromaticum* have demonstrated that enzyme inhibitory responses may vary significantly depending on plant part and extract type, further reinforcing the organ‐dependent patterns observed in the present work [4]. These literature findings do not imply equivalent potency but support the biological plausibility of the activities detected in *C. aksekiense*.

For cholinesterases, the distinct distribution of activities among the organs of *C. aksekiense* is consistent with findings from *C. aromaticum*, where Petrović et al. [4] demonstrated that both extract type (MeOH vs. essential oil) and plant part (root vs. aerial) exert a decisive influence on cholinesterase responses. Their observation that the aerial methanolic extract acted as an activator, whereas the root extract showed modest inhibition, underscores how even intraplant differences can dramatically alter ChE outcomes. Such heterogeneity coincides with the differentiated ChE‐modulating capacity observed among the leaves, roots, and stems of *C. aksekiense*, suggesting that organ‐specific metabolite allocation likely shapes cholinesterase inhibition across the genus.

Tyrosinase inhibition by *C. aksekiense* also falls within the phytochemical logic of the group. Several isolated constituents previously reported from *C. bulbosum*—notably β‐sitosterol‐3‐*O*‐β‐D‐glucopyranoside—have shown measurable tyrosinase‐modulating effects [41]. Although the tyrosinase activities in *C. aksekiense* exhibit only subtle differences among organs, the trend nonetheless parallels the modest but compound‐dependent inhibitory responses reported for *C. bulbosum* extracts and isolates. This agreement suggests that shared structural motifs across *Chaerophyllum* metabolites may contribute to the conserved, moderate tyrosinase inhibition observed in different species.

Carbohydrate‐hydrolyzing enzyme inhibition showed the clearest divergence among organs in *C. aksekiense*, a pattern strongly supported by the literature. In *C. bulbosum*, both extract classes and individual compounds display markedly selective inhibition toward α‐amylase and α‐glucosidase [36, 41]. For instance, luteolin‐7‐*O*‐β‐D‐glucopyranoside demonstrates potent α‐glucosidase inhibition while exerting only competitive or modest effects on α‐amylase [36]. The organ‐dependent selectivity in *C. aksekiense*—with roots more effective against α‐amylase and leaves/stems more responsive toward α‐glucosidase—mirrors this compound‐level specificity and indicates a comparable functional partitioning of phenolics and glycosides among plant parts.

The enzyme‐modulating behaviors of *C. aksekiense* conform to a broader pattern already recognized within the genus: Activity is strongly dependent on plant part, extraction medium, and metabolite composition. Prior studies clearly show that essential oils, methanolic extracts, isolated sterols, glycosides, and long‐chain esters from *Chaerophyllum* taxa differ sharply in their inhibitory profiles [4, 36, 41]. The present findings are therefore not only the first report for *C. aksekiense* but also a valuable contribution demonstrating that organ‐level biochemical specialization translates into distinct inhibitory signatures, particularly for carbohydrate‐hydrolyzing enzymes and cholinesterases.

### 3.4 Correlations Among Phenolic Compounds and Assays

The correlation matrix (Table 4) revealed well‐defined association patterns linking phenolic constituents with antioxidant and enzyme‐related responses. Among global antioxidant parameters, total phenolics showed strong positive correlations with CUPRAC (*r* = 0.975), DPPH (*r* = 0.866), and ABTS (*r* = 0.868), underscoring the dominant contribution of phenolic enrichment to electron‐ and radical‐based antioxidant outcomes. In contrast, total flavonoids were negatively associated with most radical‐scavenging and reducing assays but positively linked to FICA (*r* = 0.582), indicating differential functional roles of flavonoid subclasses in metal chelation versus redox‐oriented mechanisms.

**TABLE 4:** Correlations among phenolic compounds and assays.

	TAP	DPPH	ABTS	CUPRAC	FRAP	FICA	AChEIA	BChEIA	TIA	AAIA	AGIA
DPPH radical	0.658										
ABTS radical cation	0.729	0.972									
CUPRAC reducing power	0.709	0.903	0.898								
FRAP reducing power	0.516	0.972	0.932	0.798							
Ferrous ion chelating	−0.440	−0.430	−0.471	−0.768	−0.264						
RACI	0.625	0.986	0.967	0.847	0.989	−0.334					
AChE inhibition	0.889	0.635	0.703	0.807	0.453	−0.727					
BChE inhibition	0.295	−0.347	−0.277	0.015	−0.556	−0.481	0.433				
Tyrosinase inhibition	0.955	0.577	0.690	0.669	0.418	−0.509	0.913	0.391			
α‐Amylase inhibition	0.034	−0.569	−0.493	−0.201	−0.742	−0.379	0.212	0.957	0.164		
α‐Glucosidase inhibition	0.845	0.772	0.797	0.592	0.739	−0.040	0.584	−0.201	0.737	−0.454	
Total flavonoid	−0.832	−0.284	−0.358	−0.513	−0.062	0.582	−0.864	−0.758	−0.870	−0.550	−0.456
Total phenolic	0.611	0.866	0.868	0.975	0.782	−0.789	0.754	−0.050	0.570	−0.226	0.484
Hyperoside	−0.093	−0.171	−0.213	−0.534	−0.043	0.929	−0.459	−0.440	−0.206	−0.444	0.308
Chlorogenic acid	−0.934	−0.769	−0.779	−0.704	−0.662	0.270	−0.777	−0.079	−0.852	0.205	−0.935
Vanillin	−0.098	−0.758	−0.719	−0.700	−0.817	0.437	−0.225	0.578	−0.062	0.631	−0.239
Ferulic acid	−0.122	−0.454	−0.471	−0.701	−0.382	0.883	−0.445	−0.086	−0.193	−0.066	0.120
3‐Hydroxybenzoic acid	−0.532	−0.861	−0.815	−0.573	−0.918	−0.081	−0.321	0.621	−0.397	0.816	−0.865
p‐Coumaric acid	0.021	−0.678	−0.637	−0.598	−0.764	0.349	−0.093	0.648	0.061	0.668	−0.161
Quercetin	−0.688	−0.102	−0.177	−0.417	0.129	0.648	−0.776	−0.887	−0.745	−0.728	−0.229
4‐Hydroxybenzoic acid	−0.527	−0.840	−0.788	−0.544	−0.897	−0.117	−0.302	0.614	−0.386	0.812	−0.868
Syringic acid	−0.131	−0.758	−0.720	−0.744	−0.795	0.525	−0.290	0.492	−0.100	0.551	−0.216
Protocatechuic acid	−0.099	−0.643	−0.628	−0.743	−0.638	0.703	−0.344	0.269	−0.121	0.305	−0.050
Hesperidin	−0.162	−0.460	−0.481	−0.720	−0.376	0.908	−0.485	−0.136	−0.235	−0.103	0.098
Verbascoside	−0.723	−0.724	−0.756	−0.943	−0.562	0.909	−0.886	−0.327	−0.744	−0.129	−0.442
Gallic acid	0.947	0.824	0.841	0.851	0.689	−0.514	0.888	0.169	0.889	−0.111	0.849
Caffeic acid	0.932	0.745	0.777	0.873	0.575	−0.682	0.950	0.347	0.907	0.086	0.714
Rosmarinic acid	−0.295	−0.829	−0.769	−0.531	−0.933	−0.082	−0.145	0.798	−0.172	0.929	−0.678
Apigenin	−0.144	−0.677	−0.607	−0.316	−0.822	−0.313	0.057	0.890	−0.007	0.983	−0.605

*Note:* Data show the Pearson Correlation Coefficients between the parameters. TAP: total antioxidant activity by phosphomolybdenum method. AAIA, AGAI, AChEIA, BChEIA and TIA: α‐amylase, α‐glucosidase, acetylcholinesterase, butyrylcholinesterase and tyrosinase inhibition activities, respectively. ABTS and DPPH: ABTS and DPPH radical‐scavenging activities, respectively. CUPRAC and FRAP: CUPRAC‐ and FRAP‐reducing power potential; respectively.

Abbreviations: FICA, ferrous ion‐chelating activity; RACI, relative antioxidant capacity index.

At the individual‐compound level, several phenolics emerged as key drivers of assay variability. Gallic acid and caffeic acid exhibited consistently strong positive correlations with major antioxidant endpoints, including DPPH (*r* = 0.824 and 0.745), FRAP (*r* = 0.689 and 0.575), and particularly tyrosinase inhibition (*r* = 0.889 and 0.907). Conversely, compounds such as chlorogenic acid, 3‐hydroxybenzoic acid, 4‐hydroxybenzoic acid, rosmarinic acid, and apigenin demonstrated robust negative associations with radical‐scavenging and reducing assays (e.g., FRAP *r* = –0.662, –0.918, –0.897, –0.933, and –0.822, respectively), reflecting a distinct biochemical contribution relative to simpler phenolic acids.

Enzyme inhibition profiles exhibited equally structured trends. AChE inhibition correlated strongly with tyrosinase inhibition (*r* = 0.913) and with phenolics such as gallic acid (*r* = 0.888) and caffeic acid (*r* = 0.950). BChE inhibition, however, showed a weaker and more heterogeneous correlation pattern, aligning notably with α‐amylase inhibition (*r* = 0.957). Finally, FICA demonstrated consistently negative correlations with phenolic‐driven antioxidant outcomes but positive correlations with several glycosylated or methoxylated compounds (e.g., hyperoside: *r* = 0.929; ferulic acid: *r* = 0.883; verbascoside: *r* = 0.909), indicating a separate chelation‐dominated response domain.

These correlation structures collectively highlight the multifaceted interactions between phenolic profiles and bioactivity endpoints, and point to compound‐specific contributions that shape both antioxidant and enzyme‐modulatory behaviors.

## 4 Conclusions

This study provides the first comprehensive insight into the phytochemical composition and biological activities of different organs of *C. aksekiense*. The results revealed clear organ‐dependent metabolic specialization, reflected in both antioxidant and enzyme inhibitory profiles. Leaves were distinguished by higher phenolic content and stronger antioxidant activity, flowers by higher flavonoid accumulation, stems by stronger metal‐chelating ability, and roots by comparatively greater inhibition against some enzymes, particularly BChE and α‐amylase. These findings indicate that different organs of *C. aksekiense* may serve as selective sources of bioactive compounds depending on the intended application.

The observed antioxidant and enzyme inhibitory activities suggest that *C. aksekiense* has potential for further evaluation in nutraceutical, functional food, and enzyme‐targeted pharmacological research. However, the present findings are limited to in vitro assays. Future studies should therefore focus on in vivo validation, toxicity and bioavailability assessments, isolation of active constituents, and mechanistic investigations to better define the practical value of this species.

## Supporting Information

Additional supporting information can be found online in the Supporting Information section.

## Conflicts of Interest

The authors declare no conflicts of interest.

## Supporting information

Supplementary Material

## Data Availability

The data that support the findings of this study are available on request from the corresponding author. The data are not publicly available due to privacy or ethical restrictions.
